# Fascin 1 is dispensable for developmental and tumour angiogenesis

**DOI:** 10.1242/bio.20136031

**Published:** 2013-09-19

**Authors:** Yafeng Ma, Louise E. Reynolds, Ang Li, Richard P. Stevenson, Kairbaan M. Hodivala-Dilke, Shigeko Yamashiro, Laura M. Machesky

**Affiliations:** 1 Beatson Institute for Cancer Research, Garscube Estate, Switchback Road, Bearsden, Glasgow G61 1BD, UK; 2 Adhesion and Angiogenesis Laboratory, Centre for Tumour Biology, Barts Cancer Institute – a CRUK Centre of Excellence, Queen Mary University of London, Charterhouse Square, London EC1M 6BQ, UK; 3 Department of Molecular Biology and Biochemistry, Rutgers University, Piscataway, NJ 08855, USA

**Keywords:** fascin 1, Angiogenesis

## Abstract

The actin bundling protein fascin 1 is not expressed in adult epithelial tissues, but during development it is transiently expressed in many different cell types, and later in adults it is expressed in a subset of immune cells, nervous tissues, endothelial cells, smooth muscle cells and pericytes. In contrast to the wealth of knowledge about the role of fascin 1 in cancer cell migration and invasion, little is known about the involvement of fascin 1 in angiogenesis. We speculated that as angiogenesis involves migration and invasion of tissues by endothelial cells, fascin 1 might have a role in both normal and tumour angiogenesis. Here, we provide evidence that loss of fascin 1 causes relatively minor reductions to angiogenesis during embryonic, postnatal and cancerous development by examining E12.5 hindbrains, postnatal retinas and B16F0 tumour cell allografts in fascin 1-null mice. We also find that in fascin 1 null tissues, endothelial cells display reduced filopodia formation during sprouting. We thus propose that fascin 1 expression promotes angiogenesis via filopodia formation, but is largely dispensable for both normal and tumour angiogenesis.

## Introduction

Angiogenesis, the formation of new capillaries from existing vessels, is a fundamental process in development and tumour growth. It involves multiple cell types in sequential controlled steps. Various growth factors such as vascular endothelial growth factors (VEGFs) (Ruhrberg et al., 2002; Gerhardt et al., 2003; Nakayama et al., 2013), Notch pathway components (Roca and Adams, 2007) and cell adhesion molecules (Reynolds et al., 2002; Silva et al., 2008) including integrins have been reported to play crucial roles in angiogenesis and vasculogenesis.

The actin bundling protein fascin 1 is associated with the formation of actin-based cell membrane protrusions such as filopodia (Vignjevic et al., 2006) and invadopodia (Li et al., 2010; Schoumacher et al., 2010). A high expression level of fascin 1 is positively correlated with cell motility and invasiveness (Anilkumar et al., 2003; Li et al., 2010). Besides its upregulated expression in some motile progenitor cells during embryogenesis (Hayashi et al., 2008; Chae et al., 2009; Zanet et al., 2009; Ma et al., 2013) and many epithelial cancers (Machesky and Li, 2010), fascin 1 is moderately or highly expressed in endothelial cells (ECs) and mural cells in normal adult tissue and primary cell culture (Jawhari et al., 2003; Zhang et al., 2008; Hoelzle and Svitkina, 2012).

Here, we demonstrate the effect of loss of fascin 1 on angiogenesis and endothelial cell morphology with various *in vivo* angiogenesis models. Fascin 1 has been extensively studied in cancer cells and its role in promoting invasion and migration *in vitro* is well established, but its potential role in developmental angiogenesis or in tumour angiogenesis has not been explored. We suggest that fascin 1 facilitates angiogenesis via its well-known effects on filopodia formation and migration, but that overall the role of fascin in angiogenesis is not greatly limiting for development or tumour formation.

## Results and Discussion

Fascin 1-null C57BL/6 mice display partial neonatal death and retarded growth in early stages (Yamakita et al., 2009). Consistent with this previous observation, we also observed a lower survival rate in fascin 1-null mice (supplementary material Fig. S1A) and the surviving fascin 1-null pups showed retarded growth in their early life. The weight of fascin 1-null pups at day 7 and day 19 is approximately 60–90% of fascin 1^+/−^ or fascin 1^+/+^ pups (supplementary material Fig. S1B,C). Fascin 1 was reported previously to be expressed in endothelial cells, pericytes and smooth muscle cells and might be involved in the cardiovascular system (Adams, 2004). Immunofluorescence (IF) staining of tissue with isolectin B4 (BSI-B4) and fascin indicated that the endothelial layer and surrounding tissue (mural cells) in wild type aortas expressed fascin 1 whereas fascin 1-null mice had a complete loss of fascin 1 (supplementary material Fig. S1D).

### Fascin 1 loss delays embryonic brain angiogenesis

**Fig. 1.**
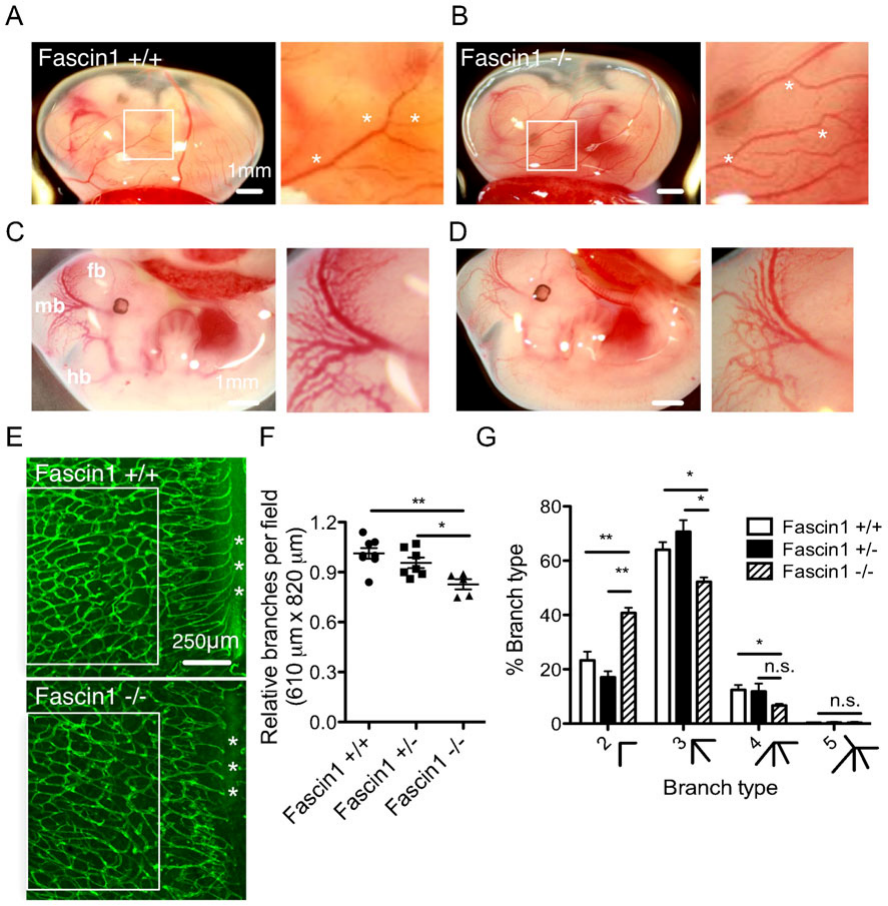
Fascin 1 deficiency reduces brain angiogenesis. (A–B) Photographs of freshly isolated embryos in intact yolk sacs and magnified areas of vessel tips in yolk sacs (E12.5, A: fascin 1^+/+^ and B: fascin 1^−/−^). White asterisks indicate vessel branch points in yolk sac. (C–D) Intact embryos and magnified midbrain area (E12.5, C: fascin 1^+/+^ or ^+/−^ and D: fascin 1^−/−^). fb, forebrain; mb, midbrain; hb, hindbrain. (E) Representative IF pictures of FITC conjugated BSI-B4 stained hindbrains (E12.5). Flat mounting the hindbrain with the ventricular side up visualizes the subventricular vascular plexus (SVP). Asterisks indicate the midline of the hindbrain. (F) Relative branch point numbers per area (610 µm×820 µm) as compared with littermate controls. 4 random areas are measured for each hindbrain. The white box in (E) is the cropped area for quantification. (G) Quantitation of branch types in fascin 1^+/+^, ^+/−^ and ^−/−^ as measured with 4× objective. 100–400 branch knots per hindbrain were examined. Results are expressed as means ± s.e.m. Mann-Whitney test, *, *P*<0.05; **, *P*<0.01 and n.s., not significant (numbers of independent hindbrain samples- fascin 1^+/+^, *n* = 7; fascin 1^+/−^, *n* = 7 and fascin 1^−/−^, *n* = 5). Bars, 1 mm (A–D), 250 µm (E).

Mouse vascular morphogenesis starts in the yolk sac on E6.5 when endothelial cells differentiate from angioblasts. By E8.5, the dorsal aortae, cardinal veins and the surrounding primitive vasculature merge. Although fascin 1-null embryos were present at the normal Mendelian ratios (supplementary material Fig. S1A) and showed no apparent hemorrhage or prenatal death (data not shown), we wondered whether non-optimal angiogenesis might contribute to abnormal brain development and retarded growth (Yamakita et al., 2009). We examined the vascular patterns in the yolk sac, midbrain and hindbrain of the developing embryos (E11.5 or E12.5) on either fresh tissue or whole-mounts stained with FITC-conjugated BS1-lectin- an EC marker. Yolk sac blood vessels showed a similar vessel pattern and network at these stages (Fig. 1A–D; supplementary material Fig. S1E,F). For quantification of vascular complexity, embryonic hindbrains are ideal tools to study the potential role of fascin in angiogenic sprouting and vascular remodeling (Fantin et al., 2013). Expression of fascin in hindbrain endothelial cells is confirmed with immunofluorescence (supplementary material Fig. S2A). Reduced branching complexity was observed in hindbrains of fascin 1^−/−^ embryos, as measured by number of branch points per area (ventricular side facing up, Fig. 1E–G, E12.5). Together these results suggest that fascin 1 plays a positive role during embryonic brain angiogenesis, but are in agreement with a previous study showing that fascin 1 is dispensable for embryonic development (Yamakita et al., 2009).

### Postnatal retinal angiogenesis is impaired in the absence of fascin 1

**Fig. 2.**
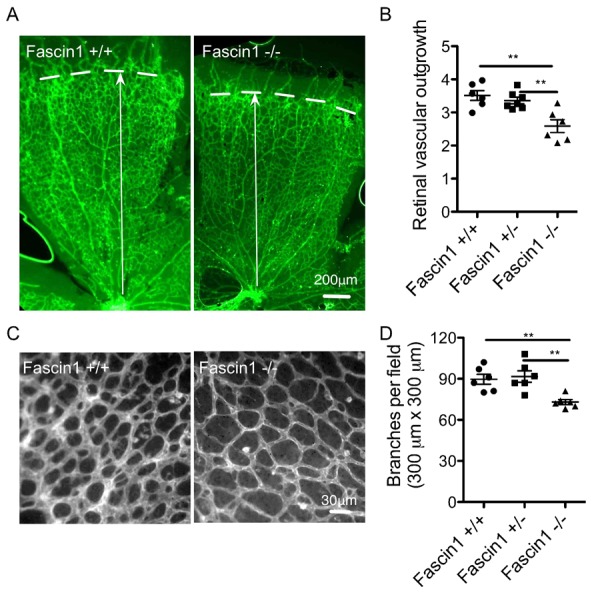
Fascin 1 deficiency restricts postnatal retinal angiogenesis. (A, C) Visualization of blood vessels by FITC conjugated BSI-B4 IF staining in fascin 1^+/+^ and fascin 1^−/−^ littermate retinas at P6. Bars, 200 µm (A), 30 µm (C). (B, D) Quantitative analysis of the retinas shows relative vessel sprout length (B), numbers of vessel branching points (300 µm×300 µm, (D). 6 retinas per genotype. 2–4 fields were measured for sprout length for each retina in (B). 2–4 fields close to vessel fronts were randomly analysed for each littermate in (C and D). Results are expressed as means ± s.e.m. Mann-Whitney test, **, *P*<0.01.

Next, we applied another widely used angiogenesis model, postnatal mouse retina, to visualize postnatal angiogenesis and vessel network patterning. Vessel sprouts emerge from the optic disc and spread perpendicularly along astrocytes and interact with macrophages (Fantin et al., 2010). We confirmed fascin expression in retina endothelial cells (supplementary material Fig. S2B). The retinal vessel network was examined for vascular sprouting at the periphery of the vessel plexus and remodeling at the center between arteries. Fascin 1^−/−^ retinas showed less radial vascular outgrowth at P6 (Fig. 2A,B). Fascin 1^−/−^ retinas also exhibited fewer branch points relative to fascin 1^+/+^ and ^+/−^ retinas (Fig. 2C,D).

### Loss of fascin 1 reduces endothelial cell filopodia formation

**Fig. 3.**
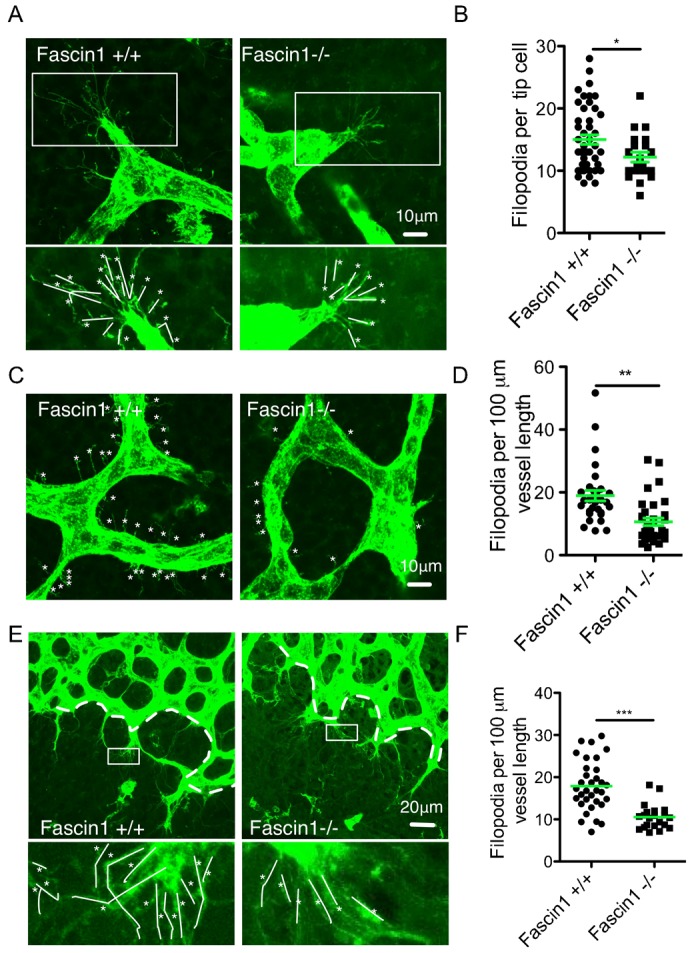
Loss of fascin 1 reduces endothelial cell filopodia in hindbrain and retinal vessels. (A–D) Representative FITC conjugated BSI-B4 stained tip cells (A) and stalk cells (C) in E12.5 hindbrain. Bars, 10 µm. (B) Filopodia per tip cells. 20–50 tip cells from 4–5 hindbrains for each genotype. (D) Quantification of filopodia per 100 µm vessel length of stalk cells. Results are means ± s.e.m., 30–35 individual areas from 4 hindbrains for each genotype. (E) Representative FITC conjugated BSI-B4 stained vessel network in P6 retinas. Zoomed-in areas show filopodia of endothelial tip cells. Bars, 20 µm. White dashed lines show angiogenic front length for quantification. (F) Filopodia per 100 µm vessel front length in retinas. 4–6 random pictures of vessel front for each littermate were counted. White asterisks mark filopodia. White lines indicate traces of filopodia. Mann-Whitney test, *, *P*<0.05; **, *P*<0.01; ***, *P*<0.001.

Tip endothelial cells (ECs) guide sprouting angiogenesis by extending long filopodia extensions in response to angiogenic factors (Gerhardt et al., 2003). These numerous actin-rich filopodia mediate EC migration and fusion (Dorrell et al., 2002; Fantin et al., 2010; Fraccaroli et al., 2012; Villefranc et al., 2013). To further examine the potential role of fascin in sprouting angiogenesis and EC filopodia extension, we analysed hindbrain tip cells and stalk cells as well as tip cells in retina sprouting fronts. Fascin 1^−/−^ hindbrain vessels generally displayed fewer filopodia extensions (supplementary material Movies 1 and 2). Tip endothelial cells in fascin^−/−^ hindbrains also exhibit fewer filopodia (Fig. 3A,B). Also, a reduction of nearly 40% in filopodia numbers per vessel length was observed in fascin 1^−/−^ hindbrains (Fig. 3C,D) as well as retinal angiogenic fronts (Fig. 3E,F). Fascin has been localized to endothelial cell filopodia (Hoelzle and Svitkina, 2012), but the role of fascin in endothelial cell filopodia extension *in vivo* has not been studied before to our knowledge. Our data agree with studies showing that dorsal root ganglion neurons and mouse embryo fibroblasts from fascin deficient mice have reduced filopodia numbers and length (Yamakita et al., 2009).

### Fascin 1 deficiency in the host reduces tumour angiogenesis, but is not limiting for tumour growth

**Fig. 4.**
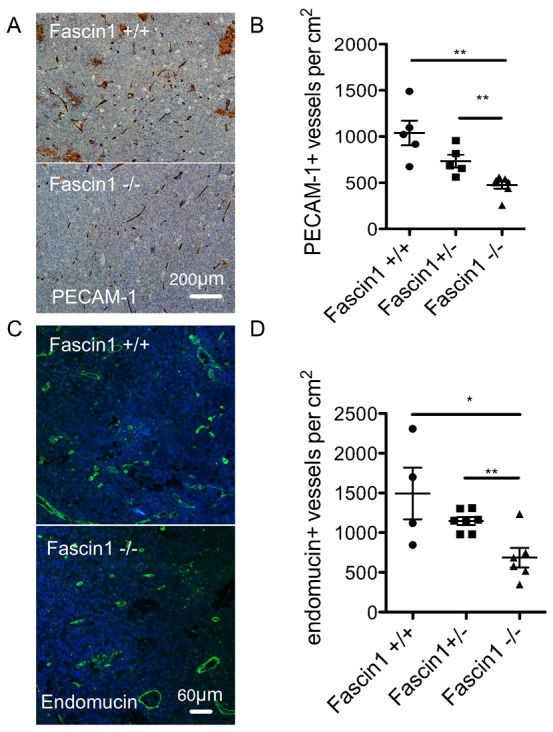
Fascin 1 deficiency reduces tumour angiogenesis. (A) Representative PECAM-1 IHC pictures with hematoxylin counter staining of B16F0 tumour sections from fascin 1^+/+^ and fascin 1^−/−^ mice and (B) quantitation of PECAM-1 labeling vessel numbers per cm^2^ tumour area. (C) Immunofluorescence staining with the blood vessel marker endomucin and (D) quantitation of endomucin labeling vessel numbers per cm^2^ tumour area. Results are means ± s.e.m., *n* = 4–7; *, *P*<0.05; **, *P*<0.01. Bars, 200 µm (A), 60 µm (C).

We next asked whether fascin 1 was important for the formation of the tumour vasculature by transplanting B16F0 melanoma cells into fascin 1-null and wild-type mice and examining subsequent tumour growth and vascularization (Reynolds et al., 2002). Fascin-1 expression in the host was not limiting for tumour growth, as tumour size and volume at 12 days after inoculation in fascin^−/−^ mice showed no obvious difference to wild type mice (supplementary material Fig. S3A). Next, we quantified the vessel density in the tumour periphery (defined as the area 1 mm close to the tumour edge) or vessel density per entire tumour area. The number of blood vessels per cm^2^ area in B16F0 allografts in fascin 1^−/−^ mice were significantly reduced compared to that in fascin 1^+/+^ and fascin 1^+/−^ mice as measured by the endothelial cell marker PECAM-1 (Platelet endothelial cell adhesion molecule-1, CD31) (Fig. 4A,B), endomucin staining (Fig. 4C,D). Adult fascin 1^+/+^ and fascin 1^−/−^ mice show similar whole blood counts (supplementary material Fig. S3B) and recruitment of macrophages and CD3 positive cells in tumours (supplementary material Fig. S3C), indicating that loss of fascin 1 in the host has no detectable effect on immune cell recruitment during angiogenesis and tumour growth. We thus speculate that while fascin expression potentiates angiogenesis, it is not essential *in vivo* and these tumours can be sufficiently vascularized to support normal growth, just as mouse development can proceed in the absence of fascin.

In summary, we show several lines of evidence to suggest the involvement of fascin 1 in angiogenesis. Fascin 1 deficiency impairs prenatal and postnatal angiogenesis, which may contribute to early growth defects observed in fascin 1-null mice. Alternatively, growth defects could be caused by fascin deficiency in other cells and lead to impaired angiogenesis. Loss of fascin 1 likely retards filopodia formation and motility during sprouting angiogenesis. Fascin 1 is well-established to promote filipodia formation in many cell types in culture and *in vivo*, but has not been previously studied in the context of endothelial tip cells or angiogenesis *in vivo*. Our observations from allografts of B16F0 melanoma also reflect that fascin contributes to, but is not limiting for tumour vascularization. As fascin inhibitors are developed with the eventual hope of targeting tumour metastasis, it is increasingly important to know what effects fascin inhibition would have on the host. We show that while fascin is abundantly expressed in endothelial and mural cells of blood vessels *in vivo*, its presence is not crucial for vasculature to form relatively normally, but can be limiting for branching and extent of vascularization. In addition to filopodia, fascin 1 also localizes in cell invasive structures, such as podosomes in endothelial cells and smooth muscle cells. Fascin 1 contributes to podosome formation, cell migration and basement membrane degradation both *in vitro* and *in vivo* in some specific organs and conditions (Varon et al., 2006; Rottiers et al., 2009; Quintavalle et al., 2010; Juin et al., 2013). Thus, the involvement of fascin 1 in the vasculature and angiogenesis is likely attributed to its function in filopodia and/or podosomes. To our knowledge, this is the first study to report that fascin 1 is required for optimal angiogenesis. Future studies could include the dissection of the role of fascin 1 in endothelial cells and mural cells and the involvement of fascin 1 in angiogenic pathways.

## Materials and Methods

### Mice

Fascin null (C57BL/6) mice (Yamakita et al., 2009) were maintained according to UK Home Office regulations. Embryonic day 0.5 was assessed at noon the day after mating and if a vaginal plug was observed, E11.5 or E12.5 embryos were isolated and judged according to their developmental stage and compared with their littermate control mice. The date of birth was regarded as postnatal day 0. To assess neonatal retinal angiogenesis, 6-day old pups from 3 litters were examined. 2–6 month old mice were used as previously described for *in vivo* tumour assays (Reynolds et al., 2002).

### Immunohistochemistry (IHC) and immunofluorescence (IF) staining

5 µm-depth sections were deparaffinised, rehydrated and treated in sodium citrate buffer (pH 6.0). After blocking with peroxidase blocking solution, samples were incubated with the following primary antibodies: rabbit anti-PECAM1 (CD31, Abcam, 1:100), mouse anti-fascin 1 (DAKO, clone 55K-2, 1:100), rabbit anti-CD3 (Vector Labs, VP-PM01, 1:75) for 1 hour. DAB-chromogenic detections were carried out using peroxidase labeled polymer (Envision kits) and detected with substrate-chromogen (Envision kits) followed with hematoxylin counterstain. For immunofluorescence staining, after deparaffinisation and rehydration, antigen retrieval using 20 µg/ml proteinase K in TE buffer (pH 8.0) (15 minutes, 37°C) was performed. After blocking, sections were incubated with the following primary antibodies (overnight, 4°C): endomucin (Santa Cruz, 1:100), rat anti-mouse F4/80 antibody (AbD Serotec, 1:50) and respective species-specific secondary antibodies (RT, 1 hour, 1:100). After PBS washes, the sections were mounted with ProLong Gold Antifade reagent with DAPI (Invitrogen).

### Hindbrain angiogenesis

E12.5 embryos were dissected as previously described (Fantin et al., 2013) and compared with their littermate control mice. For light microscopy, photographs of yolk sacs and whole embryos were taken with the same magnification at the same embryonic stage to visualize microvessel networks. The hindbrains were isolated and stained with FITC-conjugated isolectin B4 (BSI-B4, 40 µg/ml, Sigma) after 4% paraformaldehyde (PFA) fixation (4°C, overnight) and PBS-1% Triton X-100 permeabilization. The hindbrains were gently mounted and visualised on Olympus OV1000 with 10× or 60× objective (1024×1024). The branch type 2, 3, 4, 5 were defined by branch numbers and the percentages of branch types were expressed to measure the extent of angiogenesis (Stefater et al., 2011). The filopodia of the tip cells and stalk branches were analysed using z-stacked FITC-BS1-B4 positive cells and branches.

### Retinal angiogenesis

The eyes of postnatal day 6 pups were fixed in 4% PFA in PBS and washed with PBS. Retinas were dissected as previously described (Pitulescu et al., 2010; Sawamiphak et al., 2010) and permeabilised in PBT (PBS, 1% BSA and 0.5% Triton X-100) and incubated with FITC-conjugated BS1-B4 at 4°C (overnight). Retinas were mounted with ProLong Gold Antifade reagent (Invitrogen). The vessel sprout length, branch points and filopodia were measured as before (Pitulescu et al., 2010; Sawamiphak et al., 2010). The pictures were taken with a 4× objective on a stereomicroscope (Olympus BX51 FL Microscope for sprout length). Pictures were taken of the areas close to the sprout front with 20× objective for quantification of branch points. The filopodia were visualised with a 60× objective on an Olympus OV1000.

### *In vivo* tumour assays

13–16 C57BL/6 mice (2–6 month old) of fascin 1^+/+^, fascin 1^+/−^, fascin 1^−/−^ were injected subcutaneously into the scruff with 1×10^6^ of mouse melanoma B16F0 cells in 100 µl PBS. 12-days post-inoculation, the mice were sacrificed and the tumours were removed, weighed and photographed before fixation with 10% neutral formalin. Tumours were dissected and stained with hematoxylin and eosin. Size-matched tumours (6–8 per genotype) were immunostained with endothelial cell markers to quantify tumour blood vessel density. Tumour volume (v) was calculated as v = 0.5(l×w^2^), where l is tumour length (longest diameter) and w is tumour width (shortest diameter) (Reynolds et al., 2010).

### Statistical analysis

Statistical significance was calculated using the Mann-Whitney test for comparison of two groups of large datasets (fascin 1^+/+^ vs. fascin 1^−/−^) and one-way ANOVA followed by Mann-Whitney test for selected pairs of genotypes (fascin 1^+/+^, fascin 1^+/−^ and fascin 1^−/−^) (GraphPad Prism5). *P*<0.05 was considered statistically significant. *** denotes *P*<0.001, ** denotes *P*<0.01 and * denotes *P*<0.05.

## Supplementary Material

Supplementary Material
