# Supplementary material for: Fascin 1 is dispensable for developmental and tumour angiogenesis
Source: Biol Open. 2013 Sep 19;2(11):1187–91. doi: 10.1242/bio.20136031 (PMC3828765; doi:10.1242/bio.20136031)
Supplement: Supplementary Material [file supp_2_11_1187__index.html]

Fascin 1 is dispensable for developmental and tumour angiogenesis — Supplementary Material 

# Fascin 1 is dispensable for developmental and tumour angiogenesis

## bio.20136031 Supplementary Material

**Files in this Data Supplement:**

- Supplementary Material - Yafeng Ma et al. doi: 10.1242/bio.20136031
- Movie 1 - **Movie 1. 3D-projected FITC conjugated BSI-B4 stained fascin 1+/+ hindbrain vessels (211.97 µm×211.97 µm, 1024×1024, 60× objective).**
- Movie 2 - **Movie 2. 3D-projected FITC conjugated BSI-B4 stained fascin 1−/− hindbrain vessels (211.97 µm×211.97 µm, 1024×1024, 60× objective).**
