# Supplementary material for: Fascin 1 is dispensable for developmental and tumour angiogenesis
Source: Biol Open. 2013 Sep 19;2(11):1187–91. doi: 10.1242/bio.20136031 (PMC3828765; doi:10.1242/bio.20136031)
Supplement: Supplementary Material [file supp_bio.20136031_bio.20136031-s1.pdf]

Supplementary Material

Yafeng Ma et al. doi: 10.1242/bio.20136031

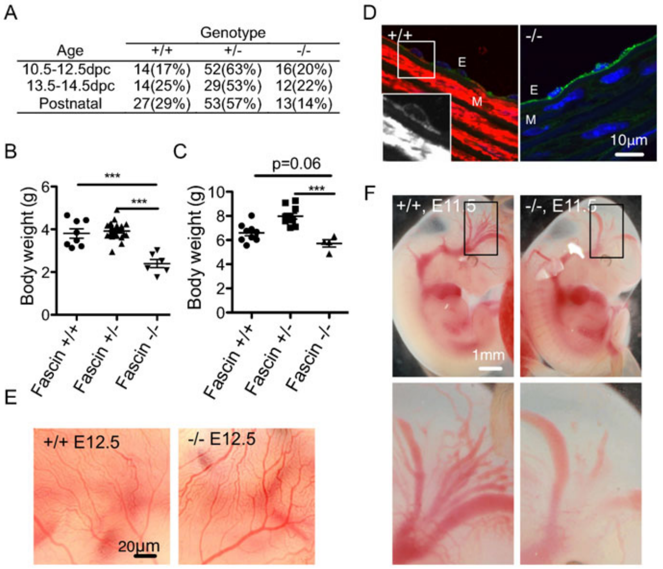

Fig. S1. (A) Genotypic analysis of progeny from fascin 1 heterozygous intercrosses (C57BL/6). Postnatal mice were scored 7 days after birth. Dpc = days postcoitum. (B–C) Body weight of fascin $1^{+/+}$, $^{+/-}$, $^{-/-}$ mice at postnatal day 7 and day 19. Results are expressed as means ± s.e.m. Mann-Whitney test, ***, $P<0.001$. (D) FITC conjugated BSI-B4 (green) and fascin 1 (red) IF stains of fascin $1^{+/+}$ and fascin $1^{-/-}$ aortas indicating a layer of endothelial cells surrounded by a few layers of mural cells. Immunostaining for fascin stains (white) in magnified area indicates that endothelial cells express lower levels of fascin compared to high level of fascin in mural cells. DAPI (blue), nuclear counterstain. E, endothelial cell layer; M, mural cell layers. (E) Representative pictures of E12.5 yolk sac showing normal vessel network in fascin $1^{-/-}$ embryos. (F) Representative pictures of E11.5 embryos showing less brain blood vessel in fascin $1^{-/-}$ embryos. Bars, 10 μm (D), 20 μm (E), 1 mm (F).

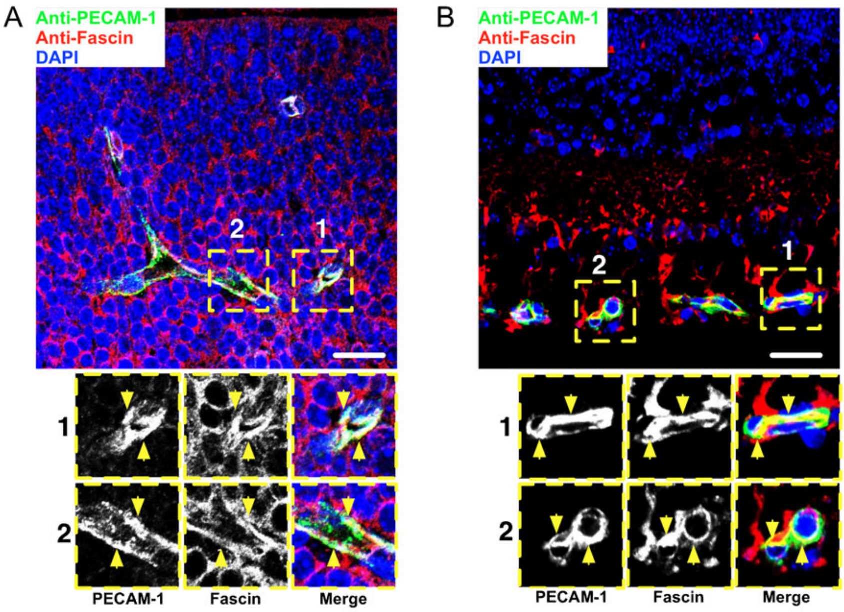

Fig. S2. (A, B) Immunostaining of hindbrain and retina tissue section with antibodies as labeled. Endothelial cells are identified with PECAM-1 staining. Insets show high-magnification views. Yellow arrows endothelial cells. Bars, 20 μm.

Biology Open

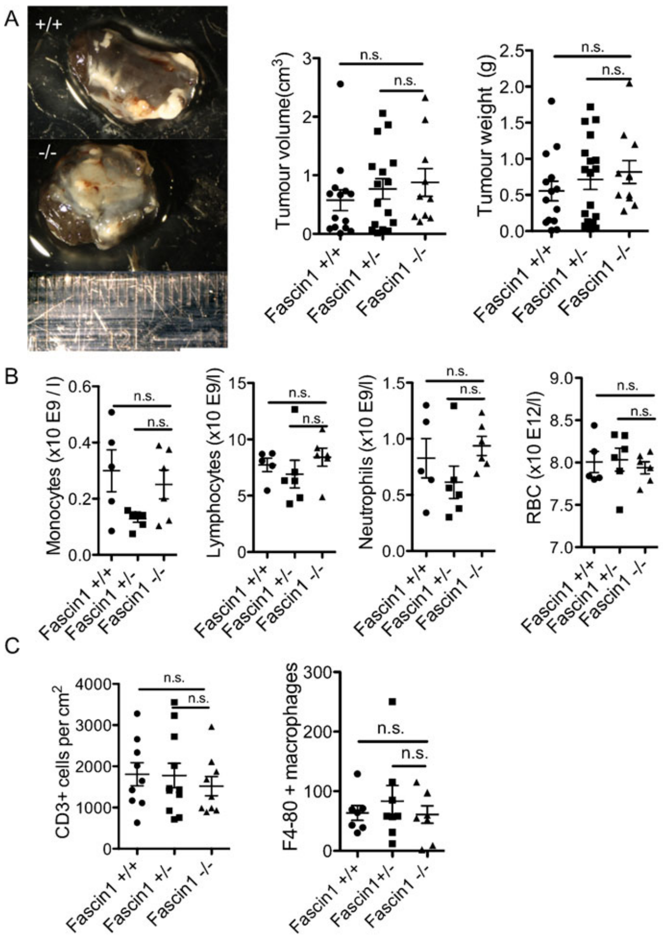

Fig. S3. (A) Representative images of B16F0 tumours from fascin $1^{+/+}$, fascin $1^{+/-}$ and fascin $1^{-/-}$ mice, tumour volume and weight as calculated in Materials and Methods (fascin $1^{+/+}$, 5; fascin $1^{+/-}$, 7; fascin $1^{-/-}$, 6). (B) Monocyte, neutrophil, lymphocyte and red blood cell (RBC) numbers of adult mice as measured by blood counts (fascin $1^{+/+}$, 5; fascin $1^{+/-}$, 6; fascin $1^{-/-}$, 6) (C) $CD3^{+}$ T cells and $F4/80^{+}$ macrophages in B16F0 tumour sections (n=7–9). Results are expressed as means ± s.e.m. Mann-Whitney test, n.s., not significant.

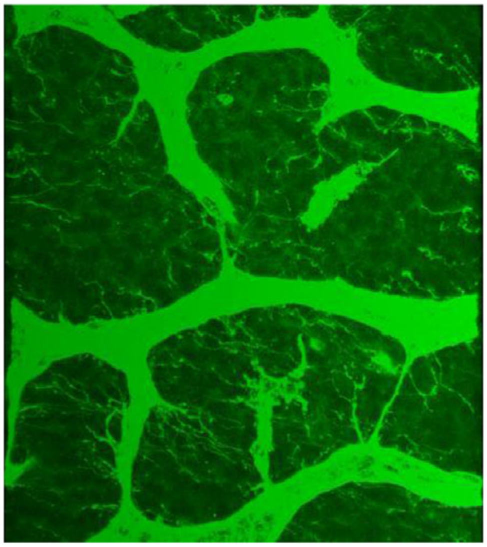

Movie 1. 3D-projected FITC conjugated BSI-B4 stained fascin $1^{+/+}$ hindbrain vessels (211.97 μm×211.97 μm, 1024×1024, 60× objective).

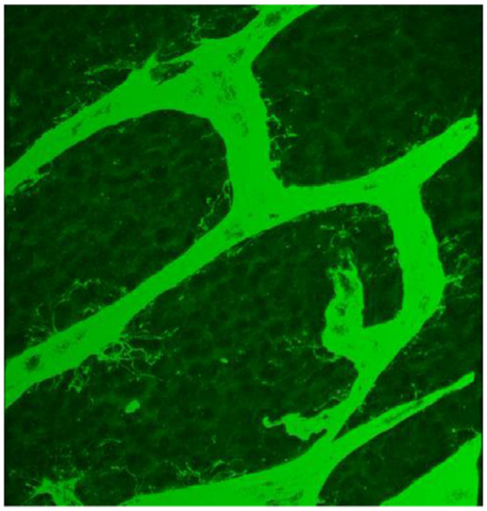

Movie 2. 3D-projected FITC conjugated BSI-B4 stained fascin $1^{-/-}$ hindbrain vessels (211.97 μm×211.97 μm, 1024×1024, 60× objective).

Biology Open
